# Retained products of conception with uterine arteriovenous malformation: A case report

**DOI:** 10.1097/MD.0000000000047781

**Published:** 2026-03-13

**Authors:** Qi Yang, Yunlang Cai

**Affiliations:** a Department of Obstetrics and Gynecology, Zhongda Hospital, School of Medicine, Southeast University, Nanjing, China.

**Keywords:** digital subtraction angiography, retained products of conception, uterine arteriovenous malformation, uterine artery embolization

## Abstract

**Rationale:**

Uterine arteriovenous malformation (UAVM) is a rare but potentially life-threatening cause of abnormal uterine bleeding. Diagnosis is particularly challenging when UAVM coexists with retained products of conception (rPOC), as both entities can present with overlapping clinical and imaging features. Accurate differentiation is essential for appropriate management, especially in women desiring preservation of fertility.

**Patient concerns:**

A woman of reproductive age presented with recurrent episodes of profuse vaginal bleeding, accompanied by progressive anemia. The severity, persistence, and recurrent nature of the hemorrhage raised strong suspicion of an underlying uterine vascular pathology, necessitating urgent diagnostic evaluation and prompt intervention.

**Diagnoses:**

Color Doppler ultrasonography and pelvic magnetic resonance imaging demonstrated a markedly hypervascular intrauterine lesion, which led to an initial presumptive diagnosis of uterine arteriovenous malformation. In the context of life-threatening hemorrhage, an acquired UAVM was considered the most likely diagnosis. However, due to substantial overlap in clinical presentation and imaging features, definitive preoperative differentiation from retained products of conception remained challenging.

**Interventions:**

Emergency uterine artery embolization was performed as a first-line, fertility-preserving therapeutic approach. Digital subtraction angiography, the diagnostic gold standard for UAVM, revealed extensive collateral vascular networks supplying the lesion, resulting in incomplete devascularization and subsequent embolization failure. Given the persistence of active bleeding and a rapid decline in hemoglobin levels, uterine-sparing surgical excision of the lesion was subsequently undertaken to achieve definitive hemostasis.

**Outcomes:**

The surgical procedure successfully controlled the hemorrhage without the need for hysterectomy. Histopathological analysis of the resected specimen demonstrated retained chorionic villi coexisting with an acquired uterine arteriovenous malformation, thereby establishing a retrospective diagnosis of rPOC-associated UAVM. The patient had an uneventful postoperative course and remained free of recurrent vaginal bleeding during follow-up.

**Lessons:**

This case illustrates the diagnostic difficulties posed by the coexistence of UAVM and rPOC, particularly due to their shared hypervascular imaging characteristics. Persistent collateral perfusion may limit the effectiveness of uterine artery embolization in such mixed lesions. Uterine-sparing surgery can provide both definitive hemostasis and histopathological confirmation when embolization fails. Early recognition of rPOC-associated UAVM is crucial for guiding individualized management and for preserving reproductive potential in affected women.

## 1. Introduction

Uterine arteriovenous malformation (UAVM) is a rare, potentially life-threatening vascular disorder characterized by direct arteriovenous communications within the myometrium, typically presenting as sudden or recurrent heavy uterine bleeding.^[1]^ Most cases are acquired, often following pregnancy-related events such as miscarriage, curettage, cesarean delivery, or retained products of conception (rPOC).^[2]^ Diagnosis is challenging because UAVM and rPOC share hypervascular features on imaging, while serum β-human chorionic gonadotropin (β-hCG) may be normal in chronic retained tissue, rendering definitive diagnosis dependent on histopathology. Management must balance hemodynamic stability, lesion vascularity, and fertility preservation. Uterine artery embolization (UAE) is first-line in reproductive-aged women, but extensive collateral circulation or coexisting rPOC may limit its effectiveness, sometimes necessitating surgical intervention.^[3]^ We report a case of rPOC-associated UAVM presenting with recurrent massive vaginal bleeding, highlighting the diagnostic challenges, limitations of embolization, and therapeutic value of uterine-sparing surgery.

## 2. Case presentation

A 34-year-old woman (G5P1) presented with a 4-day history of irregular vaginal bleeding. She reported regular menstrual cycles and no dysmenorrhea. Her history included surgical abortion with subsequent intrauterine device placement, later removed due to displacement. During her most recent menses, she developed multiple episodes of heavy bleeding with bright red blood and clots, associated with dizziness, fatigue, and mild tenesmus but no abdominal pain. External laboratory testing revealed a hemoglobin level of 88 g/L, and ultrasonography demonstrated prominent stellate myometrial blood flow. Her past surgical history included cesarean delivery for fetal distress and uterine artery embolization for a cesarean-scar pregnancy.

**Figure 1.**
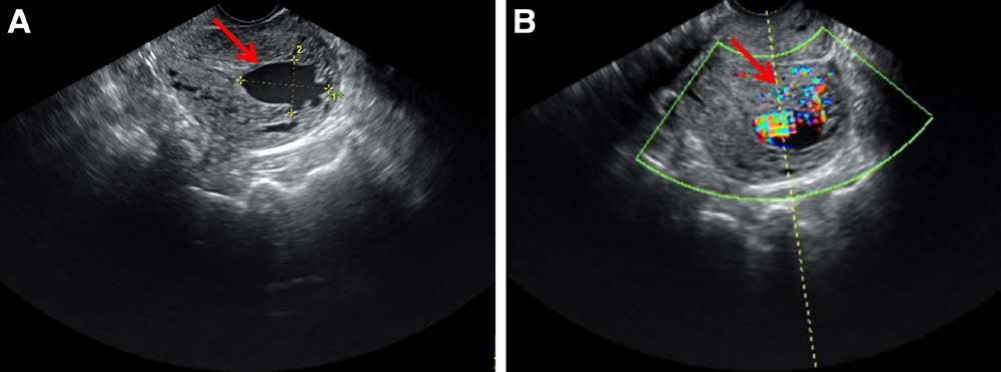
Ultrasonographic features of a uterine arteriovenous malformation. (A) Separation of the upper middle segment of the uterine cavity suggested a cystic dark area measuring 3.1 * 1.9 cm (arrow). (B) Pulsed-wave Doppler shows a mixed arteriovenous flow pattern.

**Figure 2.**
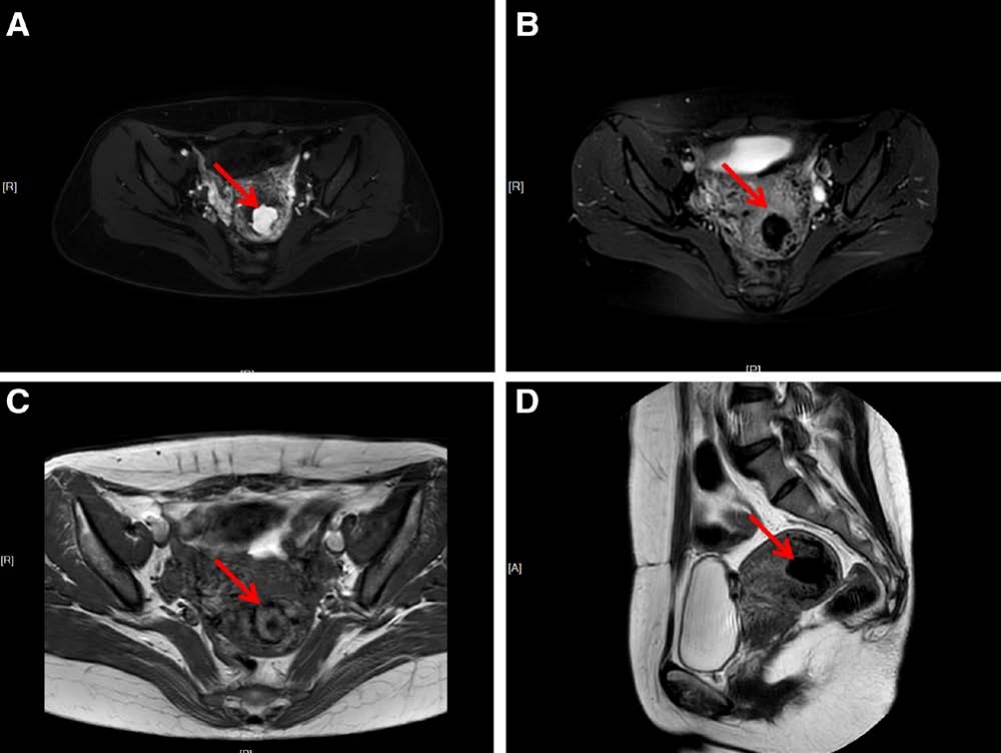
Pelvic MRI findings suggestive of an abnormal intrauterine vascular mass with possible aneurysmal formation (arrow). (A) Axial T1WI-FS image. (B) Axial T2WI-FS image. (C) Axial T1WI image. (D) Sagittal T2WI image. MRI = magnetic resonance imaging.

On admission, she was tachycardic and hypotensive, appearing anemic. Gynecological examination revealed active vaginal bleeding, an enlarged retroverted uterus with normal mobility, and unremarkable adnexa. Serum β-hCG was 0.83 mIU/mL. Transvaginal ultrasound identified a 3.1 * 1.9 cm cystic intrauterine lesion with mixed arteriovenous Doppler flow (Fig. 1). Pelvic magnetic resonance imaging showed tortuous, dilated myometrial and parametrial vessels and an abnormal intrauterine vascular mass suggestive of arteriovenous malformation with possible aneurysmal formation (Fig. 2).

**Figure 3.**
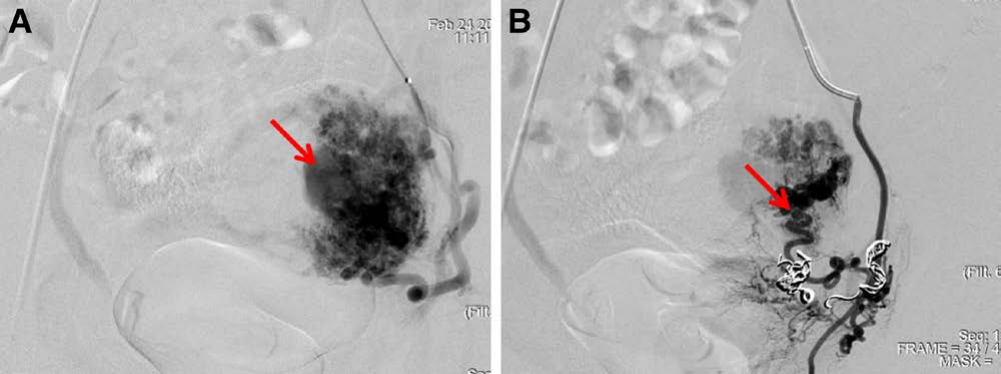
Uterine artery angiography. (A) Preoperative angiography demonstrates aneurysmal dilation and abnormal vascular pooling within the uterine artery (arrow). (B) Post-embolization imaging shows persistent and extensive collateral arterial supply (arrow).

Based on these findings, UAVM was suspected, and emergent UAE was performed. Angiography demonstrated markedly dilated, tortuous uterine arteries with multiple arteriovenous fistulas and extensive collateral supply (Fig. 3A). Despite embolization of the uterine artery trunk and collateral branches with gelatin sponge particles, significant collateral perfusion persisted, resulting in embolization failure (Fig. 3B).

**Figure 4.**
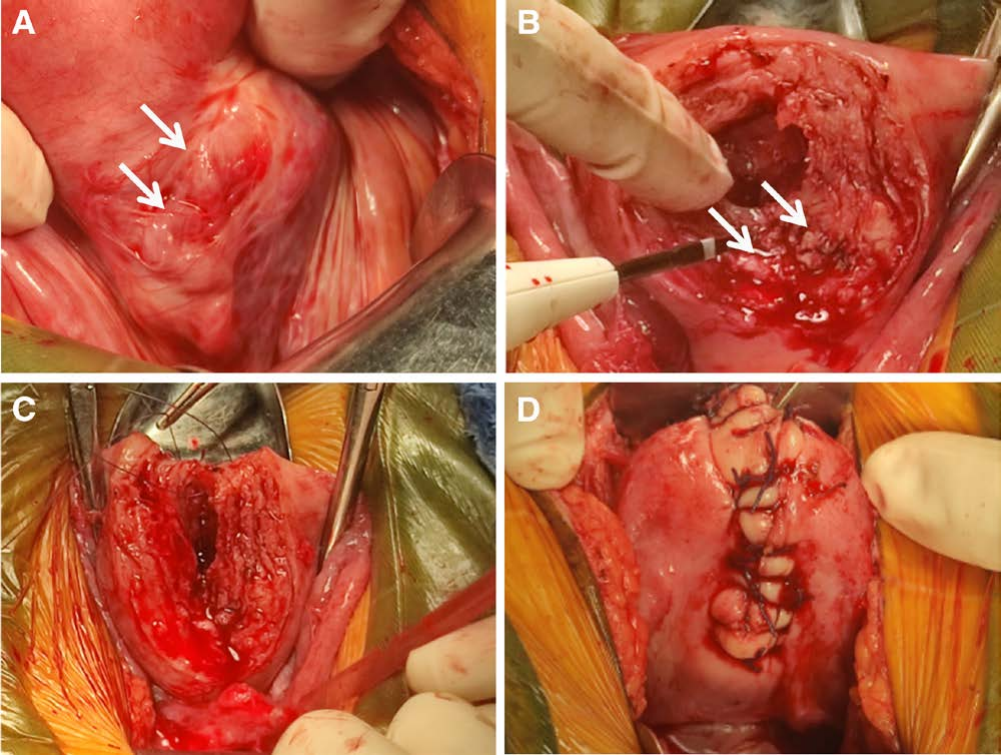
Intraoperative findings during uterine-sparing surgery. (A) Marked arterial engorgement is observed along one side of the uterus (arrow). (B) Wedge incision of the uterine wall reveals abundant vascular channels (arrow). (C) Figure-of-eight sutures used for uterine reconstruction. (D) Final appearance of the uterus after repair.

Given ongoing hemorrhage and declining hemoglobin, uterine-sparing surgery was undertaken after thorough counseling. Intraoperatively, both uterine sides exhibited rich arterial and venous vasculature (Fig. 4A). Wedge incisions in the softened anterior and posterior uterine walls exposed numerous arteriovenous channels extending to the endometrium (Fig. 4B). The affected myometrial tissue was excised, and the uterus was reconstructed with double-layer interrupted figure-of-eight sutures (Fig. 4C and D).

**Figure 5.**
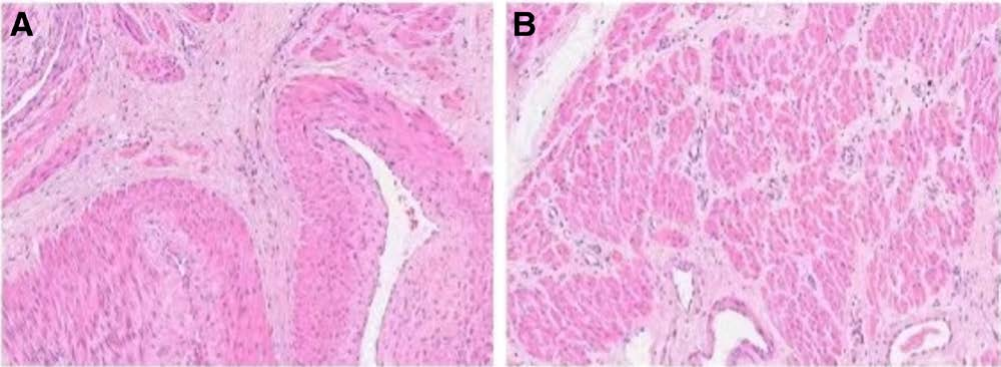
Postoperative pathology. (A) Hematoxylin and eosin (H&E) staining shows irregularly dilated, thick-walled vascular channels within the uterine myometrium, with focal intraluminal hemorrhage and thrombus formation, consistent with uterine arteriovenous fistula. (B) Organized placental chorionic villi and decidual tissue are identified within the uterine wall.

Postoperatively, vaginal bleeding decreased markedly, and the patient was discharged after 7 days. Histopathology confirmed uterine arteriovenous fistula with retained placental villi and meconium (Fig. 5). The final diagnosis was rPOC complicated by acquired UAVM. At a 1-month follow-up, the patient remained asymptomatic with no recurrent bleeding.

## 3. Discussion

UAVM is a rare vascular disorder characterized by direct arteriovenous communication within the myometrium, often presenting with sudden, profuse, and potentially life-threatening uterine bleeding.^[1]^ Although fewer than 100 cases have been documented, the true incidence is likely underestimated because UAVM frequently mimics other causes of abnormal uterine bleeding. Acquired UAVM is considerably more frequent than congenital forms and often arises secondary to pregnancy-related events, including miscarriage, curettage, cesarean delivery, and rPOC.^[4,5]^ Patients typically present with sudden or recurrent heavy uterine bleeding, which may rapidly progress to hemodynamic instability.^[2]^ In the present case, the patient experienced repeated episodes of significant vaginal bleeding accompanied by hypotension, prompting urgent evaluation for a high-flow vascular lesion.

Preoperative ultrasonography and pelvic magnetic resonance imaging in this patient revealed a markedly hypervascular intrauterine lesion with tortuous dilated vessels and high-velocity Doppler flow, consistent with the diagnosis of UAVM. Doppler ultrasonography is currently the first-line imaging modality for suspected UAVM because it can rapidly identify high-flow arteriovenous shunts.^[6]^ However, digital subtraction angiography remains the diagnostic gold standard due to its ability to delineate vascular architecture and accurately identify feeding arteries, draining veins, and collateral channels.^[7,8]^ Nevertheless, it is well established that rPOC may exhibit similar hypervascular features, and in some instances, rPOC can contribute to the formation or persistence of arteriovenous shunts through trophoblastic necrosis–induced vascular remodeling.^[9]^ This diagnostic overlap is clinically important because UAVM and rPOC may coexist, but rPOC is often only definitively diagnosed through postoperative pathological examination, as occurred in this case. The patient’s prior pregnancy termination further supports the possibility that trophoblastic remnants served as an initiating or aggravating factor in the development of UAVM.

Management of UAVM must be individualized based on hemodynamic status, severity of bleeding, lesion vascularity, and fertility preservation goals.^[1,9]^ UAE is widely regarded as the preferred first-line treatment for reproductive-aged women, as it achieves rapid hemostasis while preserving fertility.^[10,11]^ However, UAE may fail in cases with extensive collateral circulation or multiple feeding vessels. This was clearly demonstrated in our patient: despite embolization of the uterine artery and its branches, significant collateral perfusion persisted, resulting in inadequate hemostasis. The failure of UAE in this setting underscores the complexity of mixed vascular lesions and highlights that the coexistence of rPOC may sustain collateral blood flow and hinder embolization efficacy.

In recent years, high-intensity focused ultrasound (HIFU) has emerged as a novel, noninvasive therapeutic option for uterine vascular lesions, including uterine arteriovenous fistula and arteriovenous malformation. By inducing localized coagulative necrosis through focused thermal ablation, HIFU can effectively occlude abnormal arteriovenous channels while preserving surrounding myometrial tissue. A recent retrospective cohort study demonstrated that HIFU monotherapy achieved favorable hemostatic outcomes in reproductive-aged patients with uterine arteriovenous fistula, with significantly reduced intraoperative blood loss, shorter hospitalization, and lower rates of postoperative intrauterine adhesions compared with hysteroscopy-based or embolization-assisted approaches.^[12]^ Moreover, HIFU-treated patients exhibited improved reproductive outcomes during follow-up, highlighting its potential advantages in fertility preservation. These findings suggest that HIFU may represent a promising alternative or adjunctive modality for selected patients with UAVM, particularly those who are hemodynamically stable, desire future fertility, or are at risk of embolization-related complications. Nevertheless, evidence remains limited, and careful patient selection, lesion characterization, and long-term reproductive outcome assessment are essential before widespread adoption.

Given the patient’s ongoing hemorrhage, rapid hemoglobin decline, and hemodynamic vulnerability, uterine-sparing surgical excision was pursued. Surgical intervention provided definitive control of bleeding by directly removing the abnormal vascular nidus and simultaneously allowed histopathological evaluation. The postoperative pathology revealed retained chorionic villi within the excised tissue, establishing a retrospective diagnosis of rPOC coexisting with UAVM. This finding not only clarified the underlying etiology but also explains the refractory bleeding and embolization failure, as hypervascular rPOC may maintain persistent perfusion through collateral channels.^[11]^ The coexistence of UAVM with rPOC represents a particularly challenging diagnostic scenario. Serum hCG levels, often positive in rPOC, may be normal or negative in chronic retained tissue, as observed in this case, further complicating preoperative identification.^[13]^ Therefore, clinicians should maintain a high index of suspicion for rPOC in patients with recent pregnancy events, even when imaging strongly favors UAVM. Histopathological confirmation remains the diagnostic gold standard when tissue is available.

This case highlights several important clinical principles. First, UAVM and rPOC frequently share overlapping imaging features and may coexist, making exclusive reliance on imaging insufficient for definitive diagnosis. Second, embolization failure should prompt consideration of complex collateral circulation or underlying persistent gestational tissue. Third, individualized treatment strategies are essential, especially in women desiring future fertility, with emerging modalities such as HIFU offering potential benefits in carefully selected cases. Finally, surgical excision can serve as both a therapeutic and diagnostic approach when embolization is unsuccessful or diagnostic uncertainty persists.

In conclusion, the present case reinforces the importance of recognizing the interplay between UAVM and rPOC. Early and accurate identification, tailored therapeutic planning, and integration of postoperative pathological findings are essential to optimize outcomes and prevent catastrophic hemorrhage in reproductive-aged women. This case further underscores the need for clinicians to consider rPOC as a potential underlying or coexisting condition in patients presenting with suspected UAVM following pregnancy-related events.

## Author contributions

**Conceptualization:** Yunlang Cai.

**Data curation:** Qi Yang.

**Formal analysis:** Qi Yang.

**Validation:** Qi Yang.

**Visualization:** Qi Yang.

**Writing—original draft:** Qi Yang.

**Writing—review & editing:** Yunlang Cai.
